# Supplementary material for: Complex‐centric proteome profiling by SEC‐SWATH‐MS
Source: Mol Syst Biol. 2019 Jan 14;15(1):e8438. doi: 10.15252/msb.20188438 (PMC6346213; doi:10.15252/msb.20188438)
Supplement: Supplementary file 8 — Dataset EV7 [file MSB-15-e8438-s008.zip › feature_plots_string/Q15006.pdf]

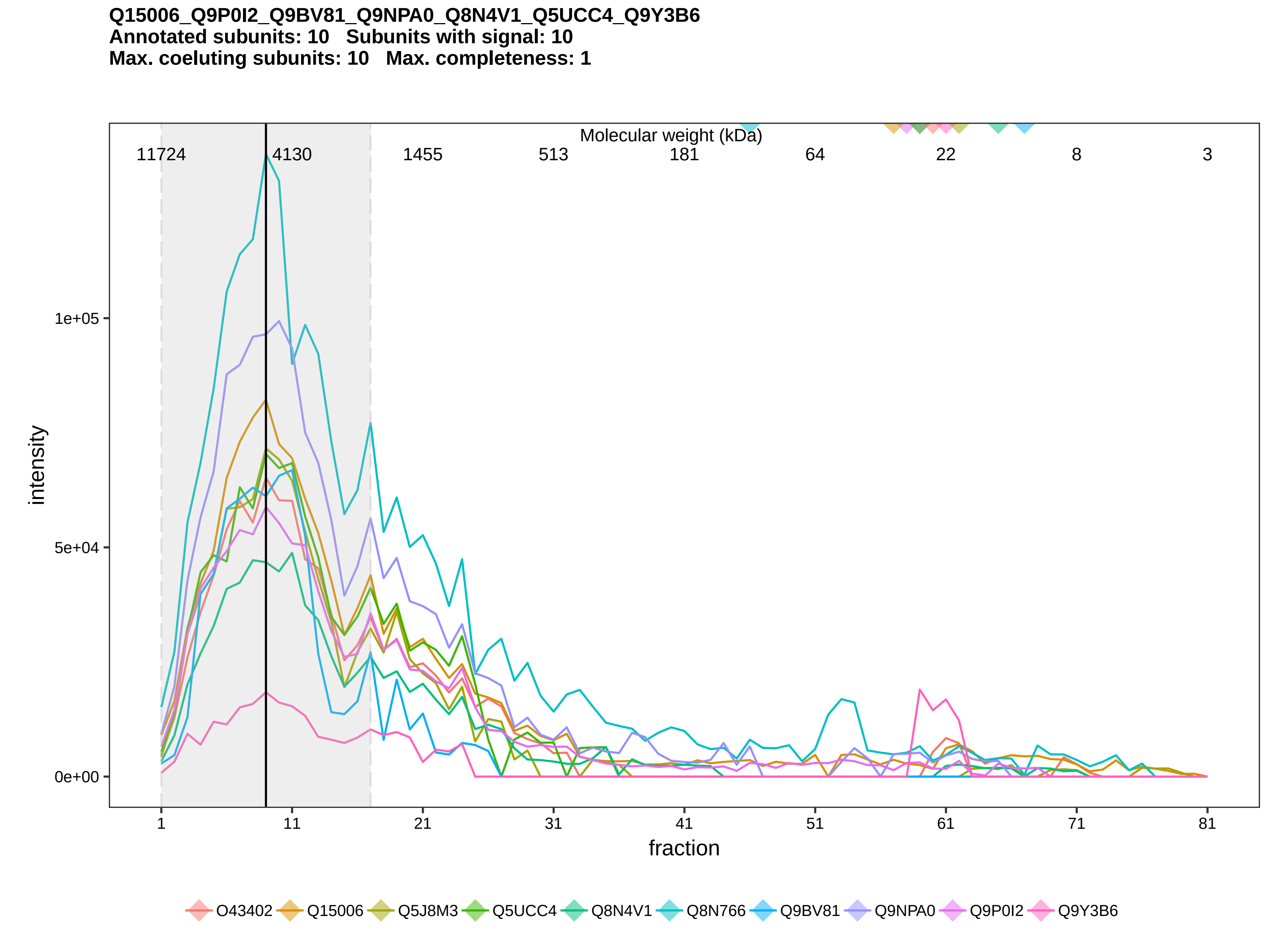
Q15006_Q9P0I2_Q9BV81_Q9NPA0_Q8N4V1_Q5UCC4_Q9Y3B6
Annotated subunits: 10 Subunits with signal: 10
Max. coeluting subunits: 10 Max. completeness: 1
Molecular weight (kDa)
11724
4130
1455
513
181
64
22
8
3
intensity
1e+05
5e+04
0e+00
1
11
21
31
41
51
61
71
81
fraction
O43402
Q15006
Q5J8M3
Q5UCC4
Q8N4V1
Q8N766
Q9BV81
Q9NPA0
Q9P0I2
Q9Y3B6
